# Enhancement of Th1-biased protective immunity against avian influenza H9N2 virus *via* oral co-administration of attenuated *Salmonella enterica* serovar Typhimurium expressing chicken interferon-α and interleukin-18 along with an inactivated vaccine

**DOI:** 10.1186/1746-6148-8-105

**Published:** 2012-07-09

**Authors:** Md Masudur Rahman, Erdenebileg Uyangaa, Young Woo Han, Seong Bum Kim, Jin Hyoung Kim, Jin Young Choi, Seong Kug Eo

**Affiliations:** 1 College of Veterinary Medicine and Bio-Safety Research Institute, Jeonju, 561-756, Republic of Korea; 2 Department of Biology, College of Natural Science, Chonbuk National University, Jeonju, 561-756, Republic of Korea

**Keywords:** Attenuated *Salmonella* vaccine, Chicken interferon-α, Chicken interleukin-18, Avian influenza H9N2, Oral delivery

## Abstract

**Background:**

Control of currently circulating re-assorted low-pathogenicity avian influenza (LPAI) H9N2 is a major concern for both animal and human health. Thus, an improved LPAI H9N2 vaccination strategy is needed to induce complete immunity in chickens against LPAI H9N2 virus strains. Cytokines play a crucial role in mounting both the type and extent of an immune response generated following infection with a pathogen or after vaccination. To improve the efficacy of inactivated LPAI H9N2 vaccine, attenuated *Salmonella enterica* serovar Typhimurium was used for oral co-administration of chicken interferon-α (chIFN-α) and chicken interleukin-18 (chIL-18) as natural immunomodulators.

**Results:**

Oral co-administration of *S. enterica* serovar Typhimurium expressing chIFN-α and chIL-18, prior to vaccination with inactivated AI H9N2 vaccine, modulated the immune response of chickens against the vaccine antigen through enhanced humoral and Th1-biased cell-mediated immunity, compared to chickens that received single administration of *S. enterica* serovar Typhimurium expressing either chIFN-α or chIL-18. To further test the protective efficacy of this improved vaccination regimen, immunized chickens were intra-tracheally challenged with a high dose of LPAI H9N2 virus. Combined administration of *S. enterica* serovar Typhimurium expressing chIFN-α and chIL-18 showed markedly enhanced protection compared to single administration of the construct, as determined by mortality, clinical severity, and feed and water intake. This enhancement of protective immunity was further confirmed by reduced rectal shedding and replication of AIV H9N2 in different tissues of challenged chickens.

**Conclusions:**

Our results indicate the value of combined administration of chIFN-α and chIL-18 using a *Salmonella* vaccine strain to generate an effective immunization strategy in chickens against LPAI H9N2.

## Background

Avian influenza viruses (AIV) of the H9N2 subtype are classified as low-pathogenicity viruses both by molecular characterization and pathotyping. Among low-pathogenicity avian influenza (LPAI) viruses, this particular subtype has attracted great concern due to its wide host range [1], chance of genetic reassortment [2] and possible avian-to-human transmission [3]. Thus, circulation of H9N2 viruses in poultry not only causes industrial losses, but also poses a potential threat to human health. Although the vaccine has been proved to be highly immunogenic in laboratory trials [4] and can prevent clinical disease and reduce viral shedding in field conditions, it cannot prevent vaccinated poultry from becoming infected and from shedding wild viruses in farm settings [5]. Therefore, an improved vaccination strategy is urgently required to control LPAI H9N2 outbreaks in poultry farms.

Cytokines are natural mediators of innate and adaptive immune responses which play a crucial role in controlling the immune system. The use of chicken cytokines is becoming more feasible with the recent cloning of a number of cytokine genes since the chicken’s immune system is similar to that of mammals [6]. Recent studies of avian cytokines identified a number of cytokines having immunomodulatory and antiviral properties against several viral infections. Additionally, some chicken cytokines have already been proven to have potent adjuvant activities [7]. In fact, the use of recombinant chicken cytokines as adjuvants is attracting extensive attention over existing oil-based or chemical adjuvants, because they promote better protection without causing any adverse site reactions or distress to chickens when administered with a vaccine. Chicken interferon-α (chIFN-α) belongs to type I IFNs and plays an essential role in the host antiviral response by stimulating the T-dependent lymphocyte system and induction of numerous IFN-stimulated genes (ISGs) [8]. Interleukin-18 (IL-18), originally known as potent interferon-γ (IFN-γ)-inducing factor (IGIF), shares properties with IL-12 and both cytokines act synergistically to promote IFN-γ production, which is well characterized as having immunomodulatory and adjuvant properties [9]. Recent studies showed that recombinant chIL-18 has immunomodulatory and anti-viral properties against AIV [10]. Therefore, both chIFN-α and chIL-18 may have great value for use either singly or in combination in disease prevention strategies in chickens. However, the practical mass administration of chicken cytokines to control poultry diseases is particularly limited due to the absence of a cost effective delivery system.

To this end, we investigated the immunomodulatory functions of chIFN-α and chIL-18 using attenuated *Salmonella enterica* serovar Typhimurium as an oral delivery vehicle. The combined oral administration of these two cytokines using attenuated *S. enterica* serovar Typhimurium modulated immune responses of chickens against inactivated AI H9N2 vaccine through enhanced humoral and Th1-biased cell-mediated immunity. Subsequently, oral co-administration of *S. enterica* serovar Typhimurium expressing chIFN-α and chIL-18 conferred better protection against a high-dose challenge of homologous AI H9N2 virus. Our results indicate the useful value of combined administration of chIFN-α and chIL-18 using attenuated *S. enterica* serovar Typhimurium in inactivated H9N2 LPAI vaccination.

## Methods

### Animals and ethics statement

SPF White Leghorn layer chickens were obtained from OrientBio (Seongnam-Si, Korea), and reared with formulated commercial feed and water provided *ad libitum* throughout the experimental period. All experimental and animal management procedures were undertaken in accordance with the requirements of the Animal Care and Ethics Committees of Chonbuk National University. The animal facility of Chonbuk National University is fully accredited by the National Association of Laboratory Animal Care.

### Cells and viruses

LPAIV H9N2 strain, A/Chicken/Korea/01310/2001 (01310), was provided by the National Veterinary Research and Quarantine Service of the Republic of Korea and used for the challenge experiments. AIV H9N2 (01310) was propagated by inoculating the allantoic cavity of 10-day-old embryonated eggs. Allantoic fluid was harvested 96 h after inoculation and the infectious viral titer was determined using 10-day-old embryonated eggs.

### Attenuated *S. enterica* serovar Typhimurium expressing chIFN-α and chIL-18

Attenuated *S. enterica* serovar Typhimurium expressing chIFN-α and chIL-18 (χ8501/chIFN-α and χ8501/chIL-18) were constructed by cloning chIFN-α and chIL-18 genes with reverse transcriptase-PCR (RT-PCR), as described elsewhere [11]. Briefly, after cloning chIFN-α and chIL-18 genes into the expression vector, pYA3560 and pYA3493, by RT-PCR, attenuated *S. enterica* serovar Typhimurium χ8501 (*hisG Δcrp-28 ΔasdA16*) was used as the host bacteria to deliver pYA3560 and pYA3493 harboring chIFN-α and chIL-18 genes by electroporation. The transformed *Salmonella* bacteria were selected after plating onto LB agar plates in the absence of diaminopimelic acid (DAP). *S. enterica* serovar Typhimurium cultures were grown at 37°C in Lennox broth, Luria-Bertani (LB) broth, or on LB agar. DAP (Sigma; 50 μg/ml) was added to induce the growth of Asd-negative bacteria [12]. The expression of chIFN-α and chIL-18 by *S. enterica* serovar Typhimurium was identified by immunoblotting following gel separation of prepared proteins by SDS-PAGE (Data not shown). Phosphate-buffered saline (PBS, pH 7.4) containing 0.01% gelatin (BSG) was used for the resuspension of *Salmonella* bacteria that were concentrated by centrifugation at 7000 × *g* at 4°C for 5 min.

### Animal experimental designs for AIV H9N2 vaccination and challenge

A total of 40 SPF chickens (32-days-old) were divided randomly into five groups. The first group (*n* = 5) was a negative control orally administered vehicle (PBS containing 0.01% gelatin) without *S. enterica* serovar Typhimurium expressing chIFN-α or chIL-18. The second group (*n* = 5) was orally administered *S. enterica* serovar Typhimurium harboring pYA3560 vector (10^9^ cfu/chicken) as a control for the empty pYA3560 vector. The remaining three groups (*n* = 10 per group), each comprising two replications (*n* = 5 per replication) for two different doses, were orally administered either *S. enterica* serovar Typhimurium expressing chIFN-α (10^9^ and 10^11^ cfu/chicken) or chIL-18 (10^9^ and 10^11^ cfu/chicken) or both in a combination (10^9^ and 10^11^ cfu/chicken). Three days after treatment, the 35-day-old chickens from all groups except the negative control, were vaccinated intramuscularly with the recommended dose of AIV H9N2 inactivated vaccine (PoulShot® Flu H9N2; Choong Ang Vaccine Inc., Daejeon, Korea). Chickens receiving a primary vaccination were boosted using the same protocol 7 days later. Blood samples were collected 7 days after the primary vaccination and 7 and 14 days after booster vaccinations followed by sera separation. Peripheral blood mononuclear cells (PBMCs) were enriched from the blood of vaccinated chickens using OptiPrepTM (13.8% iodixanol) 14 days post-booster vaccination, according to the manufacturer’s instructions (Axis-Shield, Oslo, Norway). To evaluate the protective immunity of AI H9N2 vaccine in chickens co-administered *S. enterica* serovar Typhimurium expressing chIFN-α and chIL-18, SPF chickens (7-days-old) were vaccinated according to the same protocol and intra-tracheally challenged with AIV H9N2 (01310) (10^10.83^ egg infective dose [EID]_50_/chicken) 7 days after booster vaccination at 24-days–old. Following challenge, chickens were observed daily for clinical signs and mortality throughout the duration of the experiment. Clinical signs were scored as follows: 0, no sign; 1, slight depression; 2, moderate depression + reduced movement + reduced food/water intake (anorexia); 3, moderate respiratory distress (sinusitis, cough); 4, severe respiratory distress (sinusitis, severe cough) + diarrhea; 5, death. Average feed and water intake was determined daily for 9 days after challenge. Cloacal swab samples were collected at 0, 1, 3, 5, 7, and 9 days post-infection (p.i.). Another experiment was performed using the same experimental design to collect additional samples for determination of virus amount in tissues.

### Hemagglutination inhibition (HI) assay

To determine the HI titers of the sera samples collected from vaccinated chickens, HI tests were performed with AIV H9N2 (01310) using a standard method. The geometric means of serum HI titers obtained from each group were defined as the reciprocal logarithm in a base of 2 of the highest serum dilution completely inhibiting agglutination.

### AIV H9N2 antigen-specific proliferation of PBMCs

AIV H9N2 antigen-specific proliferation of PBMCs was assessed by measuring viable cell ATP bioluminescence. Briefly, PBMCs (responder) were prepared from vaccinated chickens as previously described [13], and cultured together with stimulator cells at three different ratios. Autologous PBMCs (10^6^ cells/ml), which were isolated from corresponding chickens before vaccination and kept at liquid nitrogen tank, had been pulsed with ultraviolet (UV)-inactivated AIV H9N2 antigen (2.5×10^2^ HA units/ml) for 3 h followed by treatment with mitomycin C (25 μg/ml) for 5 min, and were employed as stimulator cells. Following 72 h incubation, the proliferated cells were evaluated using a ViaLight® Cell proliferation assay kit (Cambrex Bio Science, Rockland, ME, USA) according to the manufacturer’s instructions. PBMC stimulators that were not pulsed with UV-inactivated AIV H9N2 antigen were used for negative control.

### Real-time quantitative RT-PCR (qRT-PCR) analysis

Real-time quantitative RT-PCR (qRT-PCR) analysis was used for determining the mRNA expression levels of IFN-γ and IL-4 in PBMCs and the amount of AIV H9N2 in cloacal swab samples. Briefly, total RNA were extracted from collected samples using viral and total RNA extraction kits (iNtRON), according to the manufacturer's instructions, and then subjected to real-time qRT-PCR using a One-Step SYBR® qRT-PCR reagent kit (Takara, Shiga, Japan) and primers specific for the IFN-γ, IL-4, and AIV H9 gene (Table 1). RT and real-time PCR amplification of targeted genes were carried out under the same reaction conditions and temperature cycles as described previously [11]. After the reaction cycle was completed the temperature was increased from 50°C to 95°C at a rate of 0.2°C/15 s and fluorescence was measured every 5 s to construct a melting curve that was used to confirm the authenticity of the amplified products. A control sample that contained no template RNA was run with each assay, and qRT-PCR data was normalized using the commonly used reference gene, GAPDH (Table 1). The copy number of the experimental samples was determined by interpolating the threshold cycle values using the standard curve. All data were analyzed using CFX96^TM^ manager software version 1.6 (Bio-Rad).

**Table 1:** PCR primers for amplification of chIFN-α, chIL-18, AIH9, IFN-γ, IL-4, and GAPDH

**Target gene**		**Primer sequence (5′-3′)**	**Accession no.**	**Reference**
**chIFN-α^a^**	F	ATGGCTGTGCCTGCAAGCCCA	DQ026259.1	-
	R	CTAAGTGCGCGTGTTGCCTGT		
**chIL-18**^**b**^	F^**c**^	GAATTCGCCTTTTGTAAGGATAAAACT	HM854281.1	-
	R^**c**^	AAGCTTTCA**GTGATGGTGATGGTGATG** TAGGTTGTGCCTTTC		
**AIH9**	F	CTACTGTTGGGAGGAAGAGAATGGT	AF461510.1	[29]
	R	TGGGCGTCTTGAATAGGGTAA		
**IFN-γ**	F	CAAAGCCGCACATCAAACA	X99774	[30]
	R	TTTCACCTTCTTCACGCCATC		
**IL-4**	F	GAGAGGTTTCCTGCGTCAAG	FJ907790.1	[31]
	R	TGGTGGAAGAAGGTACGTAGG		
**GAPDH**	F	AGAACATCATCCCAGCGTCC	X01578	[30]
	R	CGGCAGGTCAGGTCAACA		

^a^The primer pair specific for chIFN-α gene was designed using chIFN-α6 nucleotide sequences (Genbank accession number DQ026259.1), and the sequences of the two primers were checked using NCBI Blast Software.

^b^The primer pair specific for chIL-18 gene was designed using chIL-18 nucleotide sequences (Genbank accession number HM854281.1), and the sequences of the two primers were checked using NCBI Blast Software.

^c^The forward and reverse primers specific for chIL-18 gene contain *Eco*RI and *Hin*dIII restriction sites as indicated by the underline. The reverse primer also contained 6×His-Tag sequences indicated in boldface.

### Statistical analysis

All data were expressed as the average ± standard error. Differences between the two groups were compared using an unpaired two-tailed Student’s *t*-test. Differences between multiple groups were compared using two-way analysis of variance (ANOVA) using SAS version 9.1 (SAS Institute Inc., Cary, NS, USA). A *p*-value < 0.05 was considered to indicate a statistically significant difference between the groups.

## Results

### Enhancement of humoral immune responses against AI vaccine by oral co-administration of *S. enterica* serovar Typhimurium expressing chIFN-a and chIL-18

**Figure 1:**
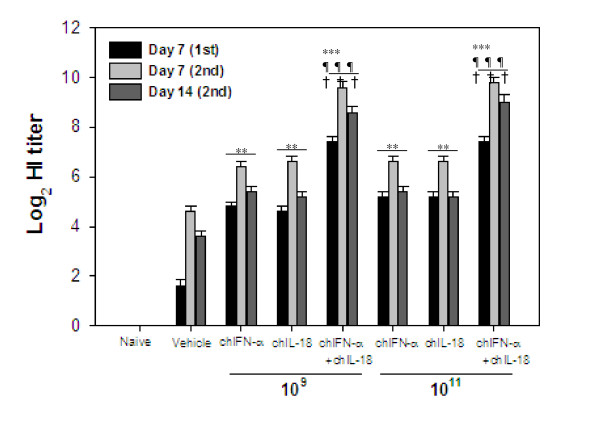
**Serum HI antibody titers of inactivated AIV-vaccinated chickens following co-administration of live attenuated*S. enterica*serovar Typhimurium expressing chIFN-α and chIL-18.** Groups of chickens were administered *S. enterica* serovar Typhimurium expressing chIFN-α and/or chIL-18 (10^9^ and 10^11^ cfu/chicken) and vaccinated with inactivated AIV H9N2 three days later. The vaccination was performed by same protocol twice at 7-day intervals. Serum samples collected from chickens of all groups 7 days after primary vaccination and 7 and 14 days after booster vaccination were subjected to HI testing. Data are expressed as reciprocal log2 of the geometric average and SEM of HI titers obtained from five chickens per group. **p < 0.01; ***p < 0.001 compared to vehicle group treated with control bacteria. ^¶¶¶^p < 0.001 compared to chIFN-α-treated chickens. ^†††^p < 0.001 compared to chIL-18-treated chickens.

In order to examine the humoral immune responses in AIV H9N2-vaccinated chickens with or without oral co-administration of *S. enterica* serovar Typhimurium expressing chIFN-α and chIL-18, groups of chickens (*n* = 5) treated with χ8501/chIFN-α or χ8501/chIL-18 or both at two different doses (10^9^ and 10^11^ cfu/chicken) were vaccinated twice with AIV H9N2 inactivated vaccine. Sera samples were collected 7 days after primary vaccination and 7 and 14 days after booster vaccination and used for the determination of HI antibody titers. Significantly enhanced HI antibody levels were observed at all three time points in the sera of both χ8501/chIFN-α- and χ8501/chIL-18-administered chickens at both doses, compared to that of χ8501 (pYA3560)-treated chickens (Figure 1). Notably, combined oral administration of χ8501/chIFN-α and χ8501/chIL-18 showed significantly enhanced HI antibody titers in the sera of AI-vaccinated chickens at both doses, compared to single administration of *S. enterica* serova Typhimurium expressing chIL-18 or chIFN-α. Therefore, these results indicate that oral co-administration of *S. enterica* serovar Typhimurium expressing chIL-18 and chIFN-α produces enhanced humoral immune responses against AI vaccine.

### Co-administration of *S. enterica* serovar typhimurium expressing chIFN-a and chIL-18 induces enhanced Th1-biased immunity against AI vaccine

**Figure 2:**
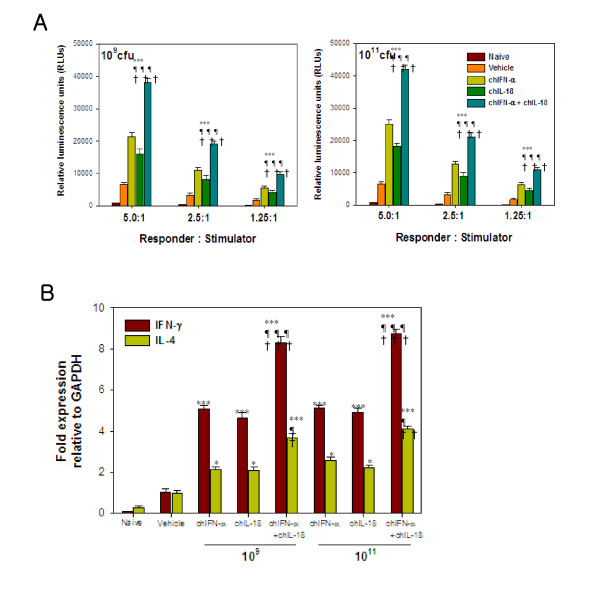
**Enhanced Th1-biased immunity in chickens that received the co-administration of*S. enterica*serovar Typhimurium expressing chIFN-α and chIL-18.** (**A**) AIV H9N2 antigen-specific proliferation of PBMCs. Groups of chickens were administered *S. enterica* serovar Typhimurium expressing chIFN-α and chIL-18 (10^9^ and 10^11^ cfu/chicken) and vaccinated with inactivated AIV H9N2 three days later. The vaccination was performed by same protocol twice at 7-day intervals. PBMCs (responders) were prepared from chickens 14 days after booster vaccination, and subsequently stimulated with naïve PBMCs (stimulators) that had been pulsed with inactivated AIV H9N2 antigen. AIV H9N2 antigen-specific proliferation of PBMCs was assessed by measuring viable cell ATP bioluminescence following incubation for 72 h. (**B**) The expression of IFN-γ and IL-4 mRNA by PBMCs following stimulation with AIV H9N2 antigen. Total RNA was extracted from PBMCs stimulated with AIV H9N2 antigen for 72 h, and subjected to real-time qRT-PCR to determine the expression of IFN-γ and IL-4. Data show the average and SEM of IFN-γ and IL-4 mRNA expression normalized to GAPDH (*n* = 5). ***p < 0.001 compared to vehicle group treated with control bacteria. p < 0.001 compared to chIFN-α-treated chickens. ^†††^p < 0.001 compared to chIL-18-treated chickens.

To evaluate cellular immune responses, PBMCs were prepared from AIV H9N2-vaccinated chickens that received oral co-administration of χ8501/chIFN-α and χ8501/chIL-18, and subsequently subjected to stimulation with autologous PBMCs that had been previously pulsed with UV-inactivated AIV H9N2 antigen. PBMCs of chickens that received χ8501/chIFN-α or χ8501/chIL-18 (10^9^ and 10^11^ cfu each per chicken) orally prior to AI vaccination were found to have significantly enhanced proliferation upon AIV H9N2 antigen-specific stimulation, compared to PBMCs from chickens that received χ8501/pYA3560 (vehicle) (Figure 2A). In particular, oral co-administration of χ8501/chIFN-α and χ8501/chIL-18 produced markedly enhanced proliferation of PBMCs upon AIV H9N2 antigen-specific stimulation than single administration of χ8501/chIFN-α or χ8501/chIL-18. In addition, the mRNA expression levels of IFN-γ and IL-4 in PBMCs were determined by real-time qRT-PCR following stimulation with AIV H9N2 antigen. Both IFN-γ and IL-4 mRNA levels in PBMCs prepared from chickens that received a single administration of χ8501/chIFN-α or χ8501/chIL-18 (10^9^ and 10^11^ cfu) were significantly enhanced, compared to chickens that received *S. enterica* serovar Typhimurium harboring empty pYA3560 vector. A markedly enhanced effect on IFN-γ and IL-4 mRNA expression in PBMCs prepared from χ8501/chIFN-α plus χ8501/chIL-18 co-administered chickens was also noticed (Figure 2B). More importantly, the expression of IFN-γ mRNA was more significantly up-regulated than IL-4 mRNA with a single administration of χ8501/chIFN-α or χ8501/chIL-18, and co-administration of χ8501/chIFN-α and χ8501/chIL-18 induced more enhanced upregulation of IFN-γ mRNA than single administration of the constructs. Taken together, our results indicate that oral co-administration of *S. enterica* serovar Typhimurium expressing chIFN-a and chIL-18 induces enhanced Th1-biased immunity against an AI vaccine, compared to single administration of *S. enterica* serovar Typhimurium expressing chIL-18 or chIFN-α in chickens.

### Enhanced protective immunity of AI vaccine by co-administration of *S. enterica* serovar Typhimurium expressing chIFN-a and chIL-18

**Figure 3:**
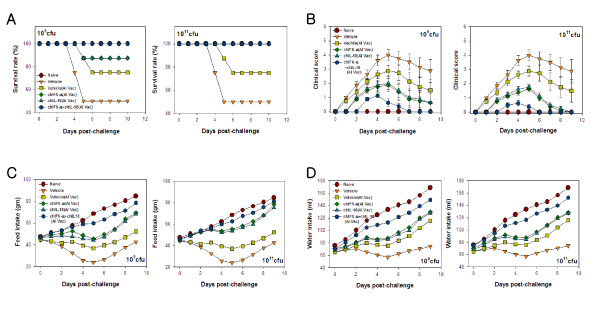
**Enhanced protective immunity of inactivated AI H9N2 vaccine by co-administration of*S. enterica*serovar Typhimurium expressing chIFN-a and chIL-18.** (**A**) Mortality of AIV H9N2-challenged chickens. Groups of chickens were administered *S. enterica* serovar Typhimurium expressing chIFN-α and chIL-18 (10^9^ and 10^11^ cfu/chicken) and vaccinated with inactivated AIV H9N2 three days later. Vaccination was performed twice at 7-day intervals. Seven days after booster vaccination, chickens were intra-tracheally infected with AIV H9N2 (10^10.83^ EID_50_/bird). Graphs show the proportion of surviving chickens on days p.i. (**B**) Clinical severity of AIV H9N2-challenged chickens. Chickens immunized with inactivated AIV H9N2 vaccine were challenged with AIV H9N2 virus and clinical severity scored daily. (**C** and **D**) Feed and water intake of AIV H9N2-challenged chickens recorded daily after AIV H9N2 challenge of inactivated AIV H9N2-vaccinated chickens. Data show the average feed (C) and water (D) intake obtained from eight chickens per group.

To evaluate the protective immunity of AI H9N2 vaccine in chickens co-administered *S. enterica* serovar Typhimurium expressing chIFN-α and chIL-18, chickens (7-days-old) co-administered *S. enterica* serovar Typhimurium expressing chIFN-α and chIL-18 were vaccinated twice at 10- and 17-days-of-age and then intra-tracheally challenged with AIV H9N2 (01310) (10^10.83^ EID_50_/chicken) 7 days after booster vaccination. Following challenge, chickens were observed daily to record mortality and clinical severity signs throughout the duration of the experiment. Mortality occurred between 4 and 6 days p.i., and the chickens that received *S. enterica* serovar Typhimurium harboring empty pYA3560 vector without AI-vaccine had the highest mortality (50%). Vaccination with inactivated AI vaccine reduced the mortality to 25% and oral administration of χ8501/chIFN-α or χ8501/chIL-18 (10^9^ cfu) prior to AI vaccination reduced it further to 12.5%. However, single administration of either χ8501/chIFN-α or χ8501/chIL-18 at a higher dose (10^11^ cfu) or combined administration at either dose (10^9^ and 10^11^ cfu) effectively protected all vaccinated chickens from mortality caused by AIV H9N2 challenge (Figure 3A). Also, when the severity of clinical signs caused by AIV H9N2 challenge infection was scored, clinical signs appeared 2 days p.i., and the severity of clinical signs peaked at 4–7 days p.i. (Figure 3B). Chickens that received single or combined administration of χ8501/chIFN-α and χ8501/chIL-18 **(**10^9^ and 10^11^ cfu) showed significant alleviation of clinical severity during the entire course of clinical infection, when compared to the group that received *S. enterica* serovar Typhimurium harboring empty pYA3560 vector (vehicle). Furthermore, average feed and water intake improved in chickens that received the AI vaccine, compared to chickens that received only empty vector (χ8501/pYA3560) (Figure 3C and D). In particular, average feed and water intake improved more when the chickens received χ8501/chIFN-α and χ8501/chIL-18 either singly or in combination (10^9^ and 10^11^ cfu) before vaccination with better result from the later. Overall, these results indicate that oral co-administration of *S. enterica* serovar Typhimurium expressing chIFN-a and chIL-18 prior to AI vaccination could markedly reduce mortality and alleviate clinical signs induced by infection with AIV H9N2. Furthermore, when we examined histopathological changes in lung and tracheal tissues of chickens 5 days after AIV H9N2 challenge infection, those that received χ8501/chIFN-α and χ8501/chIL-18 either singly or in combination (10^9^ and 10^11^ cfu) had apparently normal lungs and trachea, compared to chickens that received only empty vector without AI vaccine (data not shown). These results indicate that oral co-administration of *S. enterica* serovar Typhimurium expressing chIFN-a and chIL-18 along with AI vaccine could provide better protection against damage of lung and tracheal tissues by infection with AIV H9N2.

### Reduction of AIV H9N2 shedding and replication in vaccinated chickens

**Figure 4:**
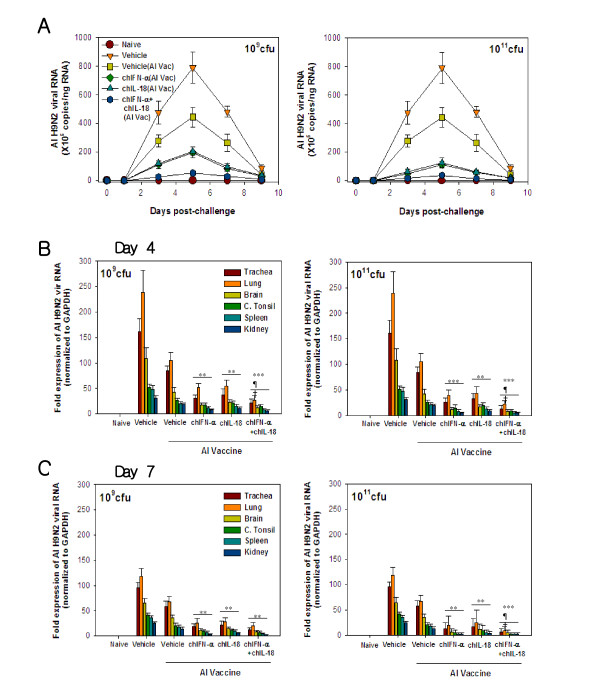
**Reduction of virus shedding and replication in AIV H9N2-challenged chickens co-administered*S. enterica*serovar Typhimurium expressing chIFN-α and chIL-18 followed by AIV H9N2 vaccination.** (**A**) Virus shedding of vaccinated chickens after AIV H9N2 challenge. Groups of chickens co-administered *S. enterica* serovar Typhimurium expressing chIFN-α and chIL-18 (10^9^ and 10^11^ cfu/chicken) followed by inactivated AIV H9N2 vaccination were intratracheally challenged with AIV H9N2 (10^10.83^ EID_50_/bird). Amounts of AIV H9N2 in cloacal swab samples taken at the indicated dates post-challenge were determined by real-time qRT-PCR using primers specific for hemagglutinin protein of AIV H9N2. Data represent the average and SEM of five chickens per group. (**B** and **C**) The amount of virus in tissues of AIV H9N2-challenged chickens. Groups of chickens that were co-administered *S. enterica* serovar Typhimurium expressing chIFN-α and chIL-18 (10^9^ and 10^11^ cfu/chicken) followed by inactivated AIV H9N2 vaccination were euthanized 4 (B) and 7 days (C) after AIV H9N2 challenge. Real-time qRT-PCR using total RNA extracted from tissues (trachea, lung, brain, cecal tonsil, spleen, and kidney) was conducted to determine AIV H9N2 amounts. Data show the average and SEM of AIV H9 fold expression obtained from four chickens per group, after normalized to GAPDH. **p < 0.01; ***p < 0.001 compared to vehicle group that was treated with control bacteria. ^¶^p < 0.05 compared to chIFN-α-treated chickens. ^†^p < 0.05 compared to chIL-18-treated chickens.

To evaluate the effect of oral co-administration of χ8501/chIFN-α and χ8501/chIL-18 on virus shedding from AIV H9N2-infected chickens that received cytokine treatment and AI vaccine, the amount of virus in cloacal swabs was determined by real-time qRT-PCR at 0, 1, 3, 5, 7 and 9 days post-challenge. Virus shedding was detected from 3 day after AIV H9N2 infection and peaked at 5 days p.i. (Figure 4A). However, the chickens that received χ8501/chIFN-α or χ8501/chIL-18 or both (10^9^ and 10^11^ cfu) before AI-vaccine had significantly lower peak levels of virus shedding at 3, 5, 7 and 9 days p.i., with a better effect from combined treatment, compared to the chickens that received only the vehicle (χ8501/pYA3560) or the vehicle plus AI vaccine. Additionally, the amount of virus in different tissues (trachea, lung, brain, cecal tonsil, spleen, and kidney) of AIV H9N2-infected chickens was determined at 4 and 7 days p.i. As expected, the amount of AIV H9N2 in different tissues of chickens that received χ8501/chIFN-α or χ8501/chIL-18 or both (10^9^ and 10^11^ cfu) prior to AI vaccination was significantly lower at both 4 (Figure 4B) and 7 days p.i. (Figure 4C), compared to chickens that received vehicle (χ8501/pYA3560). It was also noted that administration of only AI vaccine could significantly reduce the virus amounts in different tissues, compared to vehicle (χ8501/pYA3560) but significant differences existed when vehicle and AI vaccine were compared to cytokines (chIFN-α or chIL-18 or both) plus AI-vaccine, which indicates that cytokine treatment before vaccination provided better protection than vaccination alone. Taken altogether, these results indicate that oral co-administration of *S. enterica* serovar Typhimurium expressing chIFN-a and chIL-18 along with AI vaccine could alleviate clinical signs induced by AIV H9N2 infection through reduction of virus replication in tissues.

## Discussion

In the present study, we demonstrate that oral co-administration of chIFN-α and chIL-18 using attenuated *S. enterica* serovar Typhimurium modulated the immune responses of chickens against inactivated LPAI H9N2 vaccine antigen by enhancing both humoral and Th1-biased cell mediated immunity, thereby conferring better protection against homologous virus challenge. Thus, we propose that modulation of the immune response elicited by commercially available, inactivated LPAI H9N2 vaccine through combined use of chIFN-α and chIL-18 may provide a novel approach to induce complete immunity in chickens against H9N2 LPAI virus strains.

The enhanced effect of cytokine combinations has been shown empirically, based on their biological mechanisms. IFN-α and β (type I IFNs) rapidly induced by viral infection and/or a series of events have well-defined strong antiviral activity along with immunoregulatory functions. The binding of type I IFNs to type I IFN receptor complexes results in the rapid phosphorylation and activation of receptor-associated JAKs, Tyk2, and Jak1, and subsequent transcription factor STAT1/2, which induces the expression of OAS, RNase L, Mx1, and PKR genes that confer the antiviral state in cells [8]. Alternatively, IFN-γ, the only type II IFN, is a multifunctional cytokine produced primarily by T lymphocytes (Th1) and natural killer (NK) cells and IL-18 induces its production. It has been confirmed that after infection of macrophages with influenza virus, cells produce IL-18, which acts synergistically with IFN-α and enhances IFN-γ synthesis [14]. IFN-γ plays a vital role in macrophage activation and modulation of the immune system, in addition to its antiviral activity [15]. Similar to mammalian IFNs, chicken type I and type II IFNs act synergistically [16], both in terms of antiviral activity and in their ability to activate macrophages. Therefore, it is possible that chIFN-α and chIL-18 might have enhanced immunomodulatory functions when combined; however, a practical assessment of their joint function in immune modulation has not yet been addressed. It is conceivable that type II IFN-γ produced by IL-18 exposure might induce enhanced alleviation of the clinical signs of AIV H9N2 infection and modulate immunity, along with type I IFNs. Furthermore, our results are supported by the finding that chicken IL-18 cDNA linked with the recombinant encoding sequences of H5-H7 AIV in a fowl pox-based DNA vaccine (rFPV-H5-H7-IL18) successfully induced complete protection (100%) in SPF chickens after challenge with H5 AIV, and lymphocyte proliferation induced by the rFPV-H5-H7-IL18 was significantly higher than that induced by rFPV-H5-H7 alone [17]. Therefore, the present data for the first time provide valuable insight into the use of combined administration of type I IFN and IL-18 in controlling viral infection in the poultry industry.

The primary target cells for AIV infection and replication are ciliated epithelial cells. However, AIV can also infect macrophages and dendritic cells [18]. In avian species, intestinal epithelia are also targets of infection and, in the later stage of infection, mononuclear cells become involved [19]. Influenza A virus causes NS1-mediated suppression of selected genes involved in IFN and IFN-inducible gene expression [20], and induction of a weak chemokine expression in human lung epithelial cells [20], which enables the virus to replicate before the host inflammatory and antiviral responses are activated. Thus, complete protection of chickens from AIV H9N2 requires early stimulation of the immune system by immunomodulatory cytokines like chIFN-α and chIL-18. Therefore, it is possible that oral co-administration of attenuated *Salmonella* bacteria expressing chIFN-α and chIL-18 prior to vaccination with inactivated H9N2 LPAI could effectively modulate host innate and adaptive immune responses, thereby providing complete protection against AIV H9N2 challenge.

There are a few obstacles to the practical use of mass administration of cytokine proteins in livestock and poultry, such as cost, labor, and time, as well as protein stability. Establishment of a suitable delivery vector is of prime importance to enable the use of cytokines in disease prevention in this setting. Our previous report [11] and present study demonstrated the value of attenuated *Salmonella* vaccine in the oral delivery of immunomodulatory cytokines. Genetically modified *Lactococcus lactis* secreting bioactive cytokine has been found to be a useful tool for live mucosal delivery [21,22]. *L. lactis* is a safe vector for delivery of foreign genes *via* foodstuffs to the digestive tract without colonization [21,22], yet effectively induces local immune responses [23]. In contrast, live recombinant *Salmonella* vaccine can colonize gut-associated lymphoid tissue and visceral non-lymphoid and lymphoid tissues following oral administration, and subsequently stimulate local and systemic immune responses [24]. Therefore, attenuated live *Salmonella* vaccines may be a useful means to deliver bioactive cytokines to systemic as well as mucosal lymphoid tissues. Furthermore, attenuated *S. enterica* serovar Typhimuirum is a well-characterized vaccine strain available to livestock industry for the prevention of salmonellosis. A registered *Salmonella* vaccine has the potential for heterologous antigen delivery in livestock vaccination [25]. Also, since the *Salmonella* bacteria used in this study were devoid of the asd gene that is essential for a balanced-lethal host-vector system, they may have been sufficiently attenuated in their capacity to cause acute diseases in chickens and are genetically stable [26]. Accordingly, attenuated *Salmonella* vaccine expressing chIFN-α and chIL-18 produced no apparent side effect during the examination periods. However, to accomplish the control of infectious diseases in chickens effectively with a *Salmonella* delivery system, successful and prolonged colonization of *S. enterica* serovar Typhimurium expressing chIFN-α and chIL-18 may be necessary. According to previous findings, *S. enterica* serovar Typhimurium can persist in adult chickens for at least three weeks and in younger chickens up to seven weeks [27,28], but may be cleared ultimately. Therefore, it is believed that the *Salmonella* bacteria used for cytokine delivery can persist in chickens for 3-7 weeks, depending on age of chickens, and can provide continuous long term protection against virus infection.

## Conclusions

We have demonstrated that enhanced modulation of immune responses elicited by commercially available, inactivated H9N2 LPAI vaccine through combined oral administration of *S. enterica* serovar Typhimurium expressing chIFN-α and chIL-18 can protect immunized chickens from high-dose challenge of homologous virus. The results suggest that naturally occurring immunomodulatory cytokines like chIFN-α and chIL-18 can be combined with commercially available inactivated vaccines to generate an effective immunization strategy in chickens. It will be interesting to assess the protective efficacy of this immunization strategy against challenge with currently circulating heterologous virus strains in future studies.

## Abbreviations

chIFN-α, Chicken interferon-α; chIL-18, Chicken interleukin-18; DAP, Diaminopimelic acid; Asd, Aspartate β-semialdehyde dehydrogenase; HI, Hemagglutination inhibition; IGIF, Interferon-γ-inducing factor; JAK, Janus-activated kinase; LPAI, Low-pathogenocity avian influenza; PBMC, Peripheral blood monocyte; qRT-PCR, Quantitative reverse transcriptase-polymerase chain reaction; STAT, Signal transducer and activators of transcription; TCA, Trichloroacetic acid.

## Authors’ contributions

MMR: contributed during study design, collected and processed the samples, evaluated the data, wrote the manuscript. EU: supported the whole MMR’s works and wrote the manuscript. YWH and SBK: performed the real-time qRT-PCR and constructed recombinant Salmonella. JHK and JYC: contributed to collect the samples, helped with the writing of the manuscript. SKE: obtained the funding, contributed to the study design, evaluated the data, critically involved in writing manuscript. All authors read and approved the final manuscript.

## References

[B1] Alexander DJ A review of avian influenza in different bird species Vet Microbiol 2000 74 3 13 10.1016/S0378-1135(00)00160-7 10799774

[B2] Butt KM Smith GJ Chen H Zhang LJ Leung YH Xu KM Lim W Webster RG Yuen KY Peiris JS Guan Y Human infection with an avian H9N2 influenza A virus in Hong Kong in 2003 J Clin Microbiol 2005 43 5760 5767 10.1128/JCM.43.11.5760-5767.2005 16272514 PMC1287799

[B3] Li KS Xu KM Peiris JS Poon LL Yu KZ Yuen KY Shortridge KF Webster RG Guan Y Characterization of H9 subtype influenza viruses from the ducks of southern China: a candidate for the next influenza pandemic in humans? J Virol 2003 77 6988 6994 10.1128/JVI.77.12.6988-6994.2003 12768017 PMC156195

[B4] Choi JG Lee YJ Kim YJ Lee EK Jeong OM Sung HW Kim JH Kwon JH An inactivated vaccine to control the current H9N2 low pathogenic avian influenza in Korea J Vet Sci 2008 9 67 74 10.4142/jvs.2008.9.1.67 18296890 PMC2839114

[B5] Swayne DE Halvorson DA Saif YM, Barnes HJ, Glisson JR, Fadly AM, McDougald LR, Swayne DE Influenza Diseases of poultry 2003 7 Iowa State University Press, Ames 132 160

[B6] Kaiser P Advances in avian immunology-prospects for disease control: a review Avian Pathol 2010 39 309 324 10.1080/03079457.2010.508777 20954007

[B7] Asif M Jenkins KA Hilton LS Kimpton WG Bean AGD Lowenthal JW Cytokines as adjuvants for avian vaccines Immunol Cell Biol 2004 82 638 643 10.1111/j.1440-1711.2004.01295.x 15550122

[B8] Samuel CE Antiviral actions of interferons Clin Microbiol Rev 2001 14 778 809 10.1128/CMR.14.4.778-809.2001 11585785 PMC89003

[B9] Akira S The role of IL-18 in innate immunity Curr Opin Immunol 2000 12 59 63 10.1016/S0952-7915(99)00051-5 10679398

[B10] Meng S Yang L Xu C Qin Z Xu H Wang Y Sun L Liu W Recombinant chicken interferon-alpha inhibits H9N2 avian influenza virus replication in vivo by oral administration J Interferon Cytokine Res 2010 31 533 538 21323426 10.1089/jir.2010.0123

[B11] Rahman MM Uyangaa E Han YW Kim SB Kim JH Choi JY Yoo DJ Hong JT Han SB Kim B Kim K Eo SK Oral administration of live attenuated Salmonella enterica serovar Typhimurium expressing chicken interferon-α alleviates clinical signs caused by respiratory infection with avian influenza virus H9N2 Vet Microbiol 2011 154 140 151 10.1016/j.vetmic.2011.06.034 21764226

[B12] Nakayama K Kelly SM Curtiss R Construction of an asd + expression-cloning vector: stable maintenance and high level expression of cloned genes in a Salmonella vaccine strain Bio/Technology 1988 6 693 697 10.1038/nbt0688-693

[B13] Graziani-Bowering GM Graham JM Filion LG A quick, easy and inexpensive method for the isolation of human peripheral blood monocytes J. Immunol Methods 1997 207 157 168 10.1016/S0022-1759(97)00114-2 9368642

[B14] Nakanishi K Yoshimoto T Tsutsui H Okamura H Interleukin-18 regulates both TH1 and TH2 responses Ann Rev Immunol 2001 19 423 474 10.1146/annurev.immunol.19.1.423 11244043

[B15] Julkunen I Melen K Nyqvist M Pirhonen J Sareneva T Matikainen S Inflammatory responses in influenza A virus infection Vaccine 2000 19 S32 S37 11163460 10.1016/s0264-410x(00)00275-9

[B16] Moraes MP de Los Santos T Koster M Turecek T Wang H Andreyev VG Grubman MJ Enhanced antiviral activity against foot-and-mouth disease virus by a combination of type I and II porcine interferons J Virol 2007 81 7124 7135 10.1128/JVI.02775-06 17459931 PMC1933294

[B17] Mingxiao M Ningyi J Zhenguo W Ruilin W Dongliang F Min Z Gefen Y Chang L Leili J Kuoshi J Yingjiu Z Construction and immunogenicity of recombinant fowlpox vaccines coexpressing HA of AIV H5N1 and chicken IL-18 Vaccine 2006 24 4304 4311 10.1016/j.vaccine.2006.03.006 16621199

[B18] Kaufmann A Salentin R Meyer RG Bussfeld D Pauligk C Fesq H Hofmann P Nain M Gemsa D Sprenger H Defense against influenza A virus infection: essential role of the chemokine system Immunobiology 2001 204 603 613 10.1078/0171-2985-00099 11846225

[B19] Geiss GK Salvatore M Tumpey TM Carter VS Wang X Basler CF Taumenberger JK Bumgarner RE Palese P Katze MG Garcia-Sastre A Cellular transcriptional profiling in influenza A virus-infected lung epithelial cells: the role of the nonstructural NS1 protein in the evasion of the host innate defense and its potential contribution to pandemic influenza Proc Natl Acad Sci U S A 2002 99 10736 10741 10.1073/pnas.112338099 12149435 PMC125029

[B20] Veckman V Osterlund P Fagerlund R Melen K Matikainen S Julkunen I TNF-alpha and IFN-alpha enhance influenza-A-virus-induced chemokine gene expression in human A549 lung epithelial cells Virology 2006 345 96 104 10.1016/j.virol.2005.09.043 16253303

[B21] Steidler L Neirynck S Huyghebaert N Snoeck V Vermeire A Goddeeris B Cox E Remon JP Remaut E Biological containment of genetically modified Lactococcus lactis for intestinal delivery of human interleukin-10 Nat Biotechnol 2003 21 785 789 10.1038/nbt840 12808464

[B22] Rupa P Monedero V Wilkie BN Expression of bioactive porcine interferon-gamma by recombinant Lactococcus lactis Vet Microbiol 2008 129 197 202 10.1016/j.vetmic.2007.11.010 18164876

[B23] Robinson K Chamberlain LM Schofield KM Wells JM Page RWF Oral vaccination of mice against tetanus with recombinant Lactococcus lactis Nat Biotechnol 1997 15 653 657 10.1038/nbt0797-653 9219268

[B24] Medina E Guzmán CA Use of live bacterial vaccine vectors for antigen delivery: potential and limitations Vaccine 2001 19 1573 1580 10.1016/S0264-410X(00)00354-6 11166877

[B25] Bachitiar EW Sheng KC Fifis T Gamvrellis A Plebanski M Coloe PJ ZSmooker PM Delivery of a heterologous antigen by a registeredSalmonellavaccine (STM1) FEMS Microbiol Lett 2003 227 211 217 10.1016/S0378-1097(03)00683-9 14592711

[B26] Galán JE Nakayama K Curtiss R Cloning and characterization of the asd gene of Salmonella typhimurium: use in stable maintenance of recombinant plasmids in Salmonella vaccine strains Gene 1990 94 29 35 10.1016/0378-1119(90)90464-3 2227450

[B27] Beal RK Wigley P Powers C Hulme SD Barrow PA Smith AL Age at primary infection with Salmonella enterica serovar Typhimurium in the chicken influences persistence of infection and subsequent immunity to re-challenge Vet Immunol Immunopathol 2004 100 151 164 10.1016/j.vetimm.2004.04.005 15207453

[B28] Beal RK Powers C Davison TF Barrow PA Smith AL Clearance of enteric Salmonella enterica serovar Typhimurium in chickens is independent of B-cell function Infect Immun 2006 74 1442 1444 10.1128/IAI.74.2.1442-1444.2006 16428801 PMC1360334

[B29] Ong WT Omar AR Ideris A Hassan SS Development of a multiplex real-time PCR assay using SYBR Green 1 chemistry for simultaneous detection and subtyping of H9N2 influenza virus type A J Virol Methods 2007 144 57 64 10.1016/j.jviromet.2007.03.019 17512062

[B30] Niu M Han Y Li W Baculovirus up-regulates antiviral systems and induces protection against infectious bronchitis virus challenge in neonatal chicken Intl Immunopharmacol 2008 8 1609 1615 10.1016/j.intimp.2008.07.004 18707025

[B31] Xing Z Cardona CJ Li JL Dao N Tran T Andrada J Modulation of the immune responses in chickens by low-pathogenicity avian influenza virus H9N2 J Gen Virol 2008 89 1288 99 10.1099/vir.0.83362-0 18420808

